# Acute cellulitis with *Shewanella algae* bacteremia

**DOI:** 10.1590/0037-8682-0146-2023

**Published:** 2023-07-24

**Authors:** Mariana Oliveira, Pamela Ferreira, Vanessa Barcelos

**Affiliations:** 1 Hospital Divino Espírito Santo, Departamento de Medicina Interna, Ponta Delgada, Azores, Portugal. Hospital Divino Espírito Santo Departamento de Medicina Interna Ponta Delgada Azores Portugal; 2 Hospital Santo Espírito da Ilha Terceira, Departamento de Medicina Interna, Angra do Heroísmo, Azores, Portugal. Hospital Santo Espírito da Ilha Terceira Departamento de Medicina Interna Angra do Heroísmo Azores Portugal

**Keywords:** Shewanella algae, Bacteremia, Cellulitis

## Abstract

*Shewanella algae* are gram-negative bacteria commonly found in aquatic environments. Infections caused by this agent are rarely documented; however, they are increasingly reported, mainly in countries with warm to temperate climates. Herein, we present a case of a 46-year-old immunocompetent woman with acute cellulitis and *S. algae* bacteremia (the first isolation culture performed at our hospital). To better understand the epidemiology, clinical outcomes, and treatment possibilities for *S. algae* bacteremia, we searched literature for similar cases; however, we did not find any cases of infections caused by this microorganism reported in Portugal or the Azores.

## INTRODUCTION

*Shewanella algae* are saprophytic gram-negative bacilli normally found in aquatic environments (fresh water, saltwater, and sewage), as well as in raw fish^1^^-^^3^. Infections caused by this agent are rarely documented; however, they are increasingly reported^2^^,^^4^^,^^5^, with a broad spectrum of manifestations, including hepatobiliary, cutaneous, soft-tissue, respiratory, and gastrointestinal infections, along with more severe cases of sepsis and bacteremia. Moreover, endocarditis and nervous system involvement are reported^5^^,^^6^. Multiple predisposing factors are associated with *Shewanella* infections, including geographic factors (warm climates and exposure to aquatic environments) and individual risk factors (peripheral vascular disease [PVD], obesity, diabetes mellitus, hepatobiliary disease, and chronic kidney disease, especially in hemodialysis patients, neoplasms, or other immunodeficiency states). Cutaneous infections may progress to ulceration even in healthy patients.^2^^,^^5^^-^^7^. To the best of our knowledge, this is the first report on acute cellulitis with *Shewanella algae* bacteremia in Portugal, as reviewed in databases such as the Cochrane Library, LILACS, SciELO, MEDLINE, PubMed, and PubMed Central. 

## CASE REPORT

A 46-year-old woman with a history of arterial hypertension, morbid obesity (body mass index: 58 kg/m^2^), PVD, and allergy to penicillin and contrast products presented to our emergency department (ED) with inflammatory signs in the left leg for 1 week, associated with fever (maximum temperature: 39.5 °C) within the last 2 days. When asked about the epidemiological context, she denied a history of leg trauma, ingestion of shellfish or undercooked food, or exposure to aquatic environments in the previous days. Moreover, approximately 1 month ago, she presented with inflammatory signs in the same leg, without previous trauma, and was treated with amoxicillin/clavulanic acid for probable cellulitis. However, she only received the prescribed antibiotic for 3 days because she experienced an allergic reaction (an edema of the face and tongue and dyspnea); thus, the antibiotic was suspended. These skin lesions were improved with the use of antibiotic wound dressings.

**FIGURE 1:**
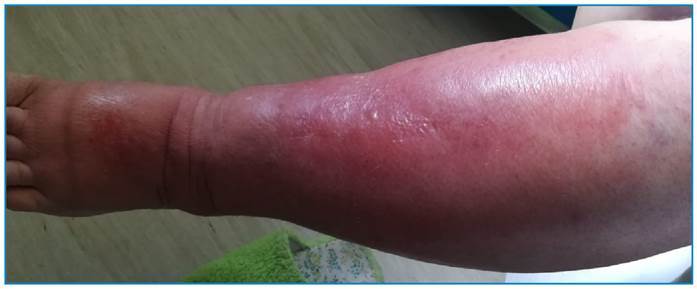
Inflammatory signs on the left lower limb upon admission - an edema (Godet grade III/IV) extending up to the knee, associated with erythema and heat, especially on the anterior surface of the leg.

Upon admission to the ED, she was alert, oriented and cooperative, eupneic on room air, hemodynamically normal, and febrile (temperature: 38.2 °C). The left lower limb (LLL) showed an exuberant edema (Godet grade III/IV) extending up to the knee/lower area of the ipsilateral thigh, associated with marked poorly circumscribed erythema and heat, especially on the anterior surface of the leg (Figure 1). Moreover, the dorsum of the foot showed an exuberant transudative edema with interdigital fissures and no apparent superinfection. Results of the remaining objective examinations were insignificant. Analytically, she showed mild leukocytosis of 12,240/µL, neutrophilia of 1,120/µL, and C-reactive protein (CRP) levels of 11.7 mg/dL. Renal function and liver enzyme levels were within normal limits. Assuming cellulitis of the LLL, we collected two pairs of blood cultures, and the patient was empirically administered with levofloxacin. In the first 48 h, inflammatory signs were markedly worsened, and the CRP level increased to 40 mg/dL; thus, we escalated to meropenem. The blood cultures isolated *S. algae*, and the susceptibility tests showed sensitivity to gentamicin, ciprofloxacin, piperacillin/tazobactam, and meropenem, with no defined pattern of resistance. The result of the human immunodeficiency virus 1/2 test was negative. The inflammatory signs in the left leg slowly regressed; however, the pain and edema were worsened. Peripheral venous ultrasonography on the LLL revealed normal permeability of the superficial and deep venous systems. Ultrasonography of the soft tissues revealed thickening and increased echogenicity of the skin and subcutaneous tissue, with no evidence of complications, such as abscesses or compartmental syndrome.

Considering the poor clinical and analytical evolution, we decided not to de-escalate the antibiotics and added empirical coverage for *Staphylococcus aureus,* initially with vancomycin (suspended after the onset of skin *rash* and pruritus) and later on, with linezolid. A complete clinical and analytical resolution was achieved with meropenem in 14 days and with linezolid in 7 days.

## DISCUSSION

Infections caused by *S. algae* have been poorly described. However, soft tissue infection is a common clinical manifestation, and chronic ulcers are one of the main underlying comorbidities^4^^,^^8^. Our patient presented with a soft tissue infection, a very common condition, and *S. algae* bacteremia (the first culture isolation performed at our hospital). This report presented a unique case because of the rarity of isolation, as the patient was an immunocompetent woman whose only risk factors were obesity and PVD without any identifiable preceding exposures. The initial clinical presentation was not atypical; the inflammatory signs of the LLL, its location and distribution, and the remaining clinical history were not suggestive of atypical pathogens. Thus, we cannot determine the point of entry with certainty. Although the patient presented with interdigital fissures, she denied having been exposed to contaminated water. However, the increasing incidence of these microorganisms requires further consideration.

The only isolated microorganism was *S. algae*. We cannot exclude the possibility that there was no co-infection or over-infection with *S. aureus* or that clinical resolution did not occur as a result of the antibiotic coverage of this organism. However, we can claim that the prolongation of the duration of administering meropenem was also a factor in the clinical improvement. Existing literature has reported more severe cases that require a longer period of receiving antibiotic therapy^5^^-^^7^. 

There are currently no guidelines for treating *Shewanella* infection*s.* Based on the available data, *S. algae* are resistant to first- and second-generation cephalosporins and penicillin, which represent the first-line treatment for soft-tissue and skin infections. Resistance to quinolones and carbapenems has already been reported^4^^,^^5^^,^^7^. The lack of guidelines and knowledge of susceptibility studies promotes the varying usage of antibiotics across centers and their prolonged use in treating the infection^6^^,^^7^^,^^9^, which may contribute to the growingly reported resistance. 

To the best of our knowledge, *S. algae* cellulitis or bacteremia has not been previously reported in Portugal, specifically in the Azorean Islands. These regions favor infections caused by *S. algae* because of their climatic conditions (hot and humid climate)^1^. Thus, this case highlights *S. algae* as a potentially emerging pathogen, even in patients without underlying comorbidities, emphasizing the need for greater case reporting to better optimize clinical decisions.
